# Benjamini-Schramm convergence of periodic orbits

**DOI:** 10.1007/s10711-022-00703-9

**Published:** 2022-08-02

**Authors:** Amir Mohammadi, Kasra Rafi

**Affiliations:** 1 Mathematics Department, UC San Diego, San Diego, CA USA; 2 Department of Mathematics, University of Toronto, Toronto, ON Canada

**Keywords:** Benjamini-Schramm convergence, Measure classification, Stabilizers, 30F60, 37A17

## Abstract

We prove a criterion for Benjamini-Schramm convergence of periodic orbits of Lie groups. This general observation is then applied to homogeneous spaces and the space of translation surfaces.

## Benjamini-Schramm convergence

Let $H\subset \mathrm{{SL}}_N(\mathbb R)$ be a non-compact semisimple group. Even though $H\subset \mathrm{{SL}}_N(\mathbb R)$, we will write *e* for the identity element in *H*. The notation *I* (for the identity matrix) will only be used when the vector space structure of the space of matrices is relevant.

Let $\Vert \;\Vert$ denote the maximum norm on $\mathrm{Mat}_N(\mathbb {R})$ with respect to the standard basis, and put

$$\begin{aligned} B^H(e,R)=\{h\in H: \Vert h-I\Vert< R\text { and }\Vert h^{-1}-I\Vert < R\}. \end{aligned}$$

We also equip *H* with the right invariant Riemannian metric induced by the Killing form (and a fixed choice of a maximal compact subgroup of *H*), and let $B^H_\mathrm{Rie}(e,r)$ denote the ball of radius *r* centered at the identity with respect to this metric. Then for every *R*, there exists $r>0$ so that

$$\begin{aligned} B^H_\mathrm{Rie}(e,r)\subset B^H(e,R). \end{aligned}$$

Let *r*(*R*) denote 1/2 the supremum of all such *r*, then $B^H_\mathrm{Rie}(e,r(R))\subset B^H(e,R)$ and $r(R)\rightarrow \infty$ as $R\rightarrow \infty$; indeed it is not difficult to see that $r(R)\ge C \log R$ where $C>0$ depends on the embedding $H\subset \mathrm{{SL}}_N(\mathbb {R})$.

Let $\Delta \subset H$ be a discrete subgroup. The injectivity radius of $y\in H/\Delta$ is define as the supremum over all $r>0$ so that the map $h\mapsto hy$ is injective on $B^H_\mathrm{Rie}(e,r)$.

Let $\Delta _n\subset H$ be a sequence of lattices in *H*. The sequence $\{H/\Delta _n: n\in \mathbb {N}\}$ *Benjamini-Schramm* converges to *H* if for every $r>0$ we have

$$\begin{aligned} \mu _n\Bigl (\{y\in H/\Delta _n: \text {injectivity radius of}\quad y<r\}\Bigr )\rightarrow 0\quad \text {as}\quad n\rightarrow \infty \end{aligned}$$

where $\mu _n$ denote the *H*-invariant probability measure on $H/\Delta _n$ for every *n*.

Throughout, we assume that *H* acts continuously on *X* preserving the measure $\mu$; also assume that $\mathrm{Stab}_H(x)$ is discrete for every $x\in X$.

An orbit $Hx\subset X$ is called *periodic* if $Hx\subset X$ is a closed subset and $\mathrm{Stab}_H(x)$ is a lattice in *H*.

For a periodic orbit *Hx*, let $\mu _{Hx}$ denote the pushforward of the *H*-invariant probability measure of $H/\mathrm{Stab}_H(x)$ to *Hx*.

### Proposition 1.1

Let $\{Hx_n: n\in \mathbb {N}\}$ be a sequence of periodic orbits in *X* satisfying that

1.1 $$\begin{aligned} \mu _{Hx_n}\rightarrow \mu \quad \text { as }\quad n\rightarrow \infty . \end{aligned}$$

Assume further that for every $R>0$ there exists a continuous function $f_R:X\rightarrow [0,\infty )$ satisfying the following two properties:

1. $$f_R(x)>0$$ for $$\mu $$-a.e. $$x\in X$$,
2. if $f_R(x)>0$ for some $x\in X$, then $\mathrm{Stab}_H(x)\cap B^H(e,R)=\{e\}$.

Then $H/\mathrm{Stab}_H(x_n)$ Benjamini-Schramm converges to *H*.

### Proof

Let $R>0$. Let $Y=Hx\subset X$ be a periodic orbit, and put $\Delta =\mathrm{Stab}(x)$. The map $h\Delta \mapsto hx$ is a homeomorphism from $H/\Delta$ onto *Y*. Let $h\Delta \in H/\Delta$, and write $y=hx\in Y$. Suppose now that $h_1h\Delta =h_2h\Delta$ for some $h_1,h_2\in B^H(e,R)$. Then $\Vert h_2^{-1}h_1-I\Vert < NR^2$ and

$$\begin{aligned} h_2^{-1}h_1\in h\Delta h^{-1}=\mathrm{Stab}_H(y). \end{aligned}$$

This and the assumption (2) in the proposition imply that

1.2 $$\begin{aligned} \text {If}\quad f_{NR^2}(y)>0,\quad \text {then the injectivity radius of}\quad h\Delta \quad \text {is at least}\quad r(R); \end{aligned}$$

recall that $$B^H_\mathrm{Rie}(e,r(R))\subset B^H(e,R)$$.

Let now $\varepsilon >0$. In view of our assumption (1) in the proposition, there exists a compact subset $K_\varepsilon \subset X$ so that

$$\begin{aligned} \mu (K_\varepsilon )>1-\varepsilon \quad \text {and}\quad f_{NR^2}(x)>0\quad \text {for all}\quad x\in K_\varepsilon . \end{aligned}$$

Since *f* is continuous and $K_\varepsilon$ is compact, there exists some $\delta >0$ so that $f_{NR^2}(x)>0$ for all $x\in \mathcal N_\delta (K_\varepsilon )$, where $\mathcal N_\delta (K_\varepsilon )$ denotes a finite open covering of the set $K_\varepsilon$ with balls of radius $\delta$ centered at points in $K_\varepsilon$.

Since $\mathcal N_\delta (K_\varepsilon )$ is an open set and $\mu _{Hx_n}\rightarrow \mu$, we conclude that

$$\begin{aligned} \liminf _n\mu _{Hx_n}\bigl (\mathcal N_\delta (K_\varepsilon )\bigr )\ge \mu \bigl (\mathcal N_{\delta /2}(K_\varepsilon )\bigr )\ge 1-\varepsilon . \end{aligned}$$

This and the fact that $\mathcal N_\delta (K_\varepsilon )\subset \{y\in Hx_n: f_{NR^2}(y)>0\}$ imply: there exists some $n_0$ so that

$$\begin{aligned} \mu _{Hx_n}\Bigl (\{y\in Hx_n: f_{NR^2}(y)>0\}\Bigr )>1-2\varepsilon \quad \text {for all}\quad n>n_0 . \end{aligned}$$

In consequence, using (1.2) we deduce that

$$\begin{aligned} \mu _{H/\mathrm{Stab}(x_{n})}\Bigl (\{y\in H/\mathrm{Stab}(x_{n}): \text {injectivity radius of}\quad y\quad \text {is}\quad< r(R)\}\Bigr )<2\varepsilon \end{aligned}$$

for all $n>n_0$. Since $r(R)\rightarrow \infty$ as $R\rightarrow \infty$, the claim follows. $\square$

In subsequent sections, we discuss two settings where Proposition 1.1 is applicable: the homogeneous setting is discussed in §2 and the space of Abelian differentials in §3; see in particular Theorems 2.2 and 3.1.

## Homogeneous spaces

Let $\mathbf{G}$ be a connencted algebraic group defined over $\mathbb {R}$, and let $G=\mathbf{G}(\mathbb {R})^\circ$ be the connected component of the identity in the Lie group $\mathbf{G}(\mathbb R)$.

Let $\Gamma \subset G$ be a lattice. Throughout this section, we assume that $\Gamma$ *is torsion free*. Let $X=G/\Gamma$, and let $\mu _X$ denote the *G*-invariant probability measure on *X*.

### Theorem 2.1

Let the notation be as above. Let $H\subset G$ be a connected semisimple Lie group. Assume that

2.1 $$\begin{aligned} \bigcap _{g\in G}gHg^{-1}\quad \text { is a finite group.} \end{aligned}$$

Let $\{Hx_n: n\in \mathbb {N}\}$ be a sequence of periodic *H*-orbits in *X* so that

1. There exists a compact subset $K\subset X$ with $Hx_n\cap K\ne \emptyset$ for all *n*.
2. For every $H\subset L\subset G$ and any closed orbit *Lx*, at most finitely many of the orbits $Hx_n$ are contained in *Lx*.

Then $H/\mathrm{Stab}_H(x_n)$ Benjamini-Schramm converges to *H*.

Note that the condition $\cap _{g\in G}gHg^{-1}$ *is a finite group* in the theorem is satisfied for instance if *G* semisimple and *H* does not contain any of the simple factors of *G*.

### Theorem 2.2

Let *M* be a real or complex hyperbolic *d*-manifold with $d\ge 3$. Assume that *M* contains infinitely many properly immersed totally geodesic hypersurfaces $\{V_n:n\in \mathbb {N}\}$. Then $\{V_n\}$ Benjamini-Schramm converges to $\mathbb H^{d-1}$ in the real hyperbolic case and to $\mathbb {C}\mathbb {H}^{d-1}$ in the complex case.

### Proof

We prove the result for the case real hyperbolic manifold, the complex case is similar.

Let $G=\mathrm{SO}(d,1)^\circ$, $\Gamma =\pi _1(M)$, and $H=\mathrm{SO}(d-1,1)^\circ$. Then $V_n$ lifts to a closed orbit $Hx_n$ in $X=G/\Gamma$ for every *n*.

Note that $H\subset G$ is a maximal connected subgroup which is not a parabolic subgroup of *G*. Therefore, the assumptions in Theorem 2.1 are satisfied for *G*, *H*, and the orbits $\{Hx_n:n\in \mathbb {N}\}$. The claim thus follows from Theorem 2.1. $\square$

We note that when $\Gamma$ is arithmetic Theorem 2.1 can be proved using the results in [1, §5]. This condition holds if $\Gamma$ is an irreducible lattice and the real rank of *G* is at least two by Margulis’ arithmeticity theorem [9]. Moreover, it was proved by Corlette and Gromov-Shoen [4, 8] that lattices in $\mathrm{SP}(n,1)$ and $F_4^{-20}$ are arithmetic. While non-arithmetic lattices in $\mathrm{SO}(n,1)$, for all *n*, and $\mathrm{SU}(n,1)$, for $n=2,3$, exist, recent developments, [2, 3, 10], show that the presence of infinitely many totally geodesic hyperplanes^1^ in real and complex hyperbolic manifolds of finite volume imply arithmeticity of their fundamental group. Therefore, in all of the interesting cases, the assertion of Theorem 2.1 can be obtained by combining rather deep existing results in the literature. However, the proof we provide here is different and is arguably simpler. In particular, our proof does not rely on the arithmeticity of $\Gamma$ and further property of congruences lattices; instead, our proof relies only on a special case of an equidistribution theorem of Mozes and Shah [14].

### Lemma 2.3

Let the notation and the assumptions be as in Theorem 2.1. Then for $\mu _X$-a.e. $x\in X$ we have

$$\begin{aligned} \mathrm{Stab}_H(x)\{e\}. \end{aligned}$$

### Proof

Let $\mathbf{H}$ denote the Zariski closure of *H* in $\mathbf{G}$. Since *H* is a connected semisimple Lie group, it has finite index in the group $H':=\mathbf{H}(\mathbb {R})\cap G$.

By Chevalley’s theorem, there exists a finite dimensional (real) representation $(\rho , W)$ of $\mathbf{G}$ and a vector $w\in W$ so that $\mathbf{H}=\{g\in \mathbf{G}: gw=w\}$. In particular, we conclude that

2.2 $$\begin{aligned} H'=G\cap \mathbf{H}=\{g\in G: gw=w\}. \end{aligned}$$

Let now $x=g_0\Gamma$. Then $\mathrm{Stab}(x)=g_0\Gamma g_0^{-1}$, and $H\cap g_0\Gamma g_0^{-1}$ is nontrivial if and only if there exists some $e\ne \gamma \in \Gamma$ so that $\gamma \in g_0^{-1}Hg_0$. Since $H\subset H'$, we conclude that $\gamma g_0^{-1}w=g_0^{-1}w$. Hence,

$$\begin{aligned} g_0^{-1}\in \mathbf{F}_\gamma =\{g\in \mathbf{G}: \gamma gw=gw\}. \end{aligned}$$

For every $\gamma \in \Gamma$, the set $\mathbf{F}_\gamma$ is an algebraic variety defined over $\mathbb {R}$. Moreover, $G=\mathbf{G}(\mathbb {R})^\circ$ is Zariski dense in $\mathbf{G}$. These and the fact that $\Gamma$ is countable imply that unless there exists some $e\ne \delta \in \Gamma$ so that

$$\begin{aligned} \delta gw=gw\quad \text {for all}\quad g\in G, \end{aligned}$$

the lemma holds — indeed in that case $G\setminus \Bigl (\cup _{\gamma \in \Gamma } \mathbf{F}_\gamma \Bigr )$ is a conull subset of *G*, and for every *g* in this set we have $\mathrm{Stab}_H(g\Gamma )=\{e\}$.

Assume now to the contrary that $G=\{g\in G: \delta gw=gw\}$ for some nontrivial $\delta \in \Gamma$. Then by (2.2) we have $\delta \in gH'g^{-1}$ for all $g\in G$, hence,

$$\begin{aligned} \delta \in \bigcap _{g\in G} gH'g^{-1}. \end{aligned}$$

Since $[H':H]<\infty$, there exists some *n* so that $\delta ^n\in gHg^{-1}$ for all $g\in G$. That is, $\delta ^n\in \cap _{g\in G}\, gHg^{-1}$. However, $\Gamma$ is torsion free and $\cap _{g\in G}\, gHg^{-1}$ is a finite group. This contradiction completes the proof. $\square$

### Proof of Theorem 2.1

We may and will assume that $G\subset \mathrm{{SL}}_N(\mathbb {R})$ for some *N*. As before, for all subgroups $L\subset G$ and all $R>0$, let

$$\begin{aligned} B^L(e, R)=\{g\in L: \Vert g-I\Vert< R \text { and }\Vert g^{-1}-I\Vert < R\} \end{aligned}$$

where $\Vert \;\Vert$ denotes the maximum norm on $\mathrm{{SL}}_N(\mathbb {R})$ with respect to the standard basis.

Recall that $\mu _X$ denotes the *G*-invariant probability measure on *X*. First note that by a theorem of Mozes and Shah [14] and our assumptions (1) and (2) in the theorem, we have

2.3 $$\begin{aligned} \mu _{Hx_n}\rightarrow \mu _X\quad \text {as}\quad n\rightarrow \infty . \end{aligned}$$

Let $\mathrm{dist}$ denote the right invariant Riemannian metric on *G* induced using the killing form. Let $R>1$, and put $\mathrm{Stab}(x)_R=\mathrm{Stab}_G(x)\cap B^G(e,R)$; this is a finite set. Define $f_R:X\rightarrow [0,\infty )$ by

$$\begin{aligned} f_R(x)=\inf \bigl \{d(h,g): h\in {B}^H(e,R), g\in \mathrm{Stab}(x)_R\setminus \{e\}\bigr \}. \end{aligned}$$

Since $\mathrm{Stab}_G(g\Gamma )=g\Gamma g^{-1}$ and *R* is fixed, $f_R$ is continuous. Furthermore, $f_R(x)>0$ for some $x\in X$ if and only if ${B}^H(e,R)\cap \mathrm{Stab}_G(x)=\{e\}$. In particular, by Lemma 2.3 we have

$$\begin{aligned} f_R(x)>0\quad \text {for}\quad \mu _X-\text {a.e.} \quad x\in X. \end{aligned}$$

Altogether, we deduce that $f_R$ satisfies the conditions in Proposition 1.1.

The theorem thus follows from Proposition 1.1 in view of (2.3). $\square$

## The space of Abelian differentials

Let $g\ge 2$, and let $\mathcal T_g$ denote the Teichmüller space of complex structure on a compact Riemann surface of genus *g*. We denote by $\mathcal M_g$ the corresponding moduli space, i.e., the quotient of $\mathcal T_g$ by the mapping class group, ${{\,\mathrm{Mod}\,}}_g$.

As it is well-known, ${{\,\mathrm{Mod}\,}}_g$ is not torsion free, however, it has subgroups of finite index which are torsion free — indeed the kernel of the natural map from ${{\,\mathrm{Mod}\,}}_g$ to $\mathrm{{Sp}}_{2g}(\mathbb Z/3\mathbb Z)$ is torsion free.

We fix, once and for all, a covering map

$$\begin{aligned} \widehat{\mathcal M}_g\rightarrow \mathcal M_g \end{aligned}$$

which corresponds to a torsion free finite index subgroup of ${{\,\mathrm{Mod}\,}}_g$.

Let $f:\mathbb {H}^2\rightarrow \mathcal M_g$ be an isometric immersion for the Teichmüller metric. Typically, $f(\mathbb {H}^2)$ is dense in $\mathcal M_g$, however, there are situations where $f(\mathbb {H}^2)$ is an algebraic curve in $\mathcal M_g$. In the latter case, the stabilizer $\Delta$ of *f* is a lattice in $\mathrm{Isom}(\mathbb {H}^2)$, and we obtain a *Teichmüller curve*

$$\begin{aligned} f:V=\mathbb {H}^2/\Delta \rightarrow \mathcal M_g. \end{aligned}$$

For every $g\ge 2$, the moduli space $\mathcal M_g$ contains a dense family of Teichmüller curves which arise as branched cover of flat tori. There are also examples of infinite families of *primitive* Teichmüller curves, i.e., Teichmüller curves which do not arise as a branched cover of flat tori, in $\mathcal M_g$ when $g=2,3,4$, [12, 13].

### Theorem 3.1

Let $\{V_n:n\in \mathbb {N}\}$ be an infinite family of Techimüller curves in $\mathcal M_g$. For every *n*, let $\widehat{V}_n\rightarrow V_n$ be a lift of $V_n$ to $\widehat{\mathcal M}_g$. Then $\{\widehat{V}_n:n\in \mathbb {N}\}$ Benjamini-Schramm converges to $\mathbb H^2$.

C. Leininger and A. Wright (independently) have supplied an alternative (and arguably softer) proof of Theorem 3.1. This argument relies on the fact that the length of shortest geodesic on Teichmüller curves tends to infinity, see Proposition 3.2, and is independent of measure classification theorems. We also thank T. Gelander for helpful communications regarding IRSs.

Here, we present a proof based on Proposition 1.1 and [6] to highlight a unifying theme between the homogeneous setting and the setting at hand.

For every $M\in \mathcal T_g$, let $\Omega (M)$ be the *g*-dimensional space of holomorphic 1-forms on *M*. By integrating a non-zero form $\omega \in \Omega (M)$ we obtain, away from the zeros of $\omega$, a flat metric $|\omega |$ on *M* and local charts whose transition functions are translations.

Form a vector bundle over the Teichmüller space $\mathcal T_g$ where the fiber over each point is $\Omega (M)$. Let $\Omega \mathcal T_g\rightarrow \mathcal T_g$ be the complement of the zero section of this vector bundle.

There is a natural action of $\mathrm{GL}^+_2(\mathbb R)$ (and hence of $\mathrm{{SL}}_2(\mathbb R)$) on $\Omega \mathcal T_g$: given a holomorphic 1-form $\omega =\mathfrak {R}(\omega )+i\mathfrak I(\omega )$ and $$h =\begin{pmatrix} a &{} b \\ c &{} d \end{pmatrix} \in \mathrm{GL}^+_2(\mathbb R)$$,

3.1 $$\begin{aligned} h\cdot \omega =\begin{pmatrix} i \\ i \end{pmatrix}\begin{pmatrix} a &{} b \\ c &{} d \end{pmatrix}\begin{pmatrix} \mathfrak {R}(\omega ) \\ \mathfrak I(\omega ) \end{pmatrix}. \end{aligned}$$

We let $\Omega \mathcal M_g\rightarrow \mathcal M_g$ denote the quotient of $\Omega \mathcal T_g$ by action of the mapping class group of $S_g$.

For every $\alpha =(\alpha _1,\ldots ,\alpha _{m})$ with $\sum \alpha _i=2g-2$, let $\mathcal H(\alpha )$ denote the set of $(M,\omega )\in \Omega \mathcal M_g$ where $\omega$ has zeros of type $\alpha$. Then $\Omega \mathcal M_g=\bigsqcup \mathcal H(\alpha )$.

Let $(M,\omega )\in \mathcal H(\alpha )$ and let $\Sigma \subset M$ denote the set of zeroes of $\omega$. Let $\{ \gamma _1, \dots , \gamma _k\}$ denote a $\mathbb Z$-basis for the relative homology group $H_1(M,\Sigma , \mathbb Z)$. (It is convenient to assume that the basis is obtained by extending a symplectic basis for the absolute homology group $H_1(M,\mathbb Z)$.) We can define a map $\Phi : \mathcal H(\alpha ) \rightarrow \mathbb C^k$ by

$$\begin{aligned} \Phi (M,\omega ) = \left( \int _{\gamma _1} \omega , \dots , \int _{\gamma _k} w \right) \end{aligned}$$

The map $\Phi$ (which depends on a choice of the basis $\{ \gamma _1, \dots , \gamma _k\}$) is a local coordinate system on $(M,\omega )$. Alternatively, we may think of the cohomology class $[\omega ] \in H^1(M,\Sigma , \mathbb C)$ as a local coordinate on the stratum $\mathcal H(\alpha )$. We will call these coordinates *period coordinates*.

The area of a translation surface is given by

$$\begin{aligned} a(M,\omega ) = \frac{i}{2} \int _M \omega \wedge \bar{\omega }. \end{aligned}$$

We let $\Omega _1\mathcal M_g$ and $\mathcal H_1(\alpha )$ denote the locus of unit area 1-forms in $\Omega \mathcal M_g$ and $\mathcal H(\alpha )$, respecitively.

### The $\mathrm{{SL}}_2(\mathbb R)$-action and the Kontsevich-Zorich cocycle

The action in (3.1) descends to an action of $\mathrm{{SL}}_2(\mathbb R)$ on $\mathcal H_1(\alpha )$. Indeed, write $\Phi (M,\omega )$ as a $2 \times d$ matrix *x*. The action of $\mathrm{{SL}}_2(\mathbb R)$ in these coordinates is linear.

Let ${{\,\mathrm{Mod}\,}}(M,\Sigma )$ be the mapping class group of *M* fixing each zero of $\omega$. We choose a fundamental domain for the action of ${{\,\mathrm{Mod}\,}}(M,\Sigma )$, and think of the dynamics on the fundamental domain. Then, the $\mathrm{{SL}}_2(\mathbb R)$ action becomes

3.2 $$\begin{aligned} x = \begin{pmatrix} \mathfrak {R}(\omega ) \\ \mathfrak I(\omega ) \end{pmatrix} \mapsto hx = \begin{pmatrix} a &{} b \\ c &{} d \end{pmatrix} \begin{pmatrix} \mathfrak {R}(\omega ) \\ \mathfrak I(\omega ) \end{pmatrix} A(h,x), \end{aligned}$$

where $A(h,x) \in \mathrm{{Sp}}_{2g}(\mathbb Z) \ltimes \mathbb Z^{m-1}$ is the *Kontsevich-Zorich cocycle*.

Thus, *A*(*h*, *x*) is the change of basis one needs to perform to return the point *hx* to the fundamental domain. It can be interpreted as the monodromy of the Gauss-Manin connection (restricted to the orbit of $\mathrm{{SL}}_2(\mathbb R)$).

### Affine measures and manifolds

For a subset $\mathcal E \subset \mathcal H_1(\alpha )$ we write

$$\begin{aligned} \mathbb R\mathcal E = \{ (M, t \omega ) : (M,\omega ) \in \mathcal E, t \in \mathbb R\} \subset \mathcal H(\alpha ). \end{aligned}$$

An ergodic $\mathrm{{SL}}_2(\mathbb R)$-invariant probability measure $\nu$ on $\mathcal H_1(\alpha )$ is called *affine* if the following hold:

(i) The support $\mathcal M$ of $\nu$ is an *immersed submanifold* of $\mathcal H_1(\alpha )$, i.e., there exists a manifold $\mathcal N$ and a proper continuous map $f: \mathcal N\rightarrow \mathcal H_1(\alpha )$ so that $\mathcal M= f(\mathcal N)$. The self-intersection set of $\mathcal M$, i.e., the set of points of $\mathcal M$ which do not have a unique preimage under *f*, is a closed subset of $\mathcal M$ of $\nu$-measure 0. Furthermore, each point in $\mathcal N$ has a neighborhood *U* such that locally $\mathbb Rf(U)$ is given by a complex linear subspace defined over $\mathbb R$ in the period coordinates.
(ii) Let $\bar{\nu }$ be the measure supported on $\mathbb R\mathcal M$ so that $d\bar{\nu }= d\nu da$. Then each point in $\mathcal N$ has a neighborhood *U* such that the restriction of $\bar{\nu }$ to $\mathbb Rf(U)$ is an affine linear measure in the period coordinates on $\mathbb Rf(U)$, i.e., it is (up to normalization) the restriction of the Lebesgue measure to the subspace $\mathbb Rf(U)$.

A suborbifold $\mathcal M$ for which there exists a measure $\nu$ such that the pair $(\mathcal M, \nu )$ satisfies (i) and (ii) is said to be *affine invariant submanifold*.

We sometimes write $\nu _\mathcal M$ to indicate the affine invariant measure $\nu$ on affine invariant submanifold $\mathcal M$.

Note that in particular, any affine invariant submanifold is a closed subset of $\mathcal H_1(\alpha )$ which is invariant under the action of $\mathrm{{SL}}_2(\mathbb R)$, and which in period coordinates is an affine subspace. We also consider the entire stratum $\mathcal H_1(\alpha )$ to be an (improper) affine invariant submanifold.

### Typical affine stabilizer is trivial

In this section, we prove the following statement:

#### Proposition 3.2

Let $(\mathcal M,\nu )\subset \mathcal H_1(\alpha ) \subset \Omega \widehat{\mathcal M}_{g,n}$ be an affine invariant submanifold. Assume that $\mathcal M$ is *not* a Teichmüller curve. Then for $\nu$-a.e. $x\in \mathcal M$,

$$\begin{aligned} \mathrm{Stab}_{\mathrm{{SL}}_2(\mathbb R)}(x) \end{aligned}$$

is trivial.

Recall that the set of self-intersections $\mathcal M'$ of $\mathcal M$ is a proper closed invariant submanifold of $\mathcal M$, hence, $\dim \mathcal M'<\dim \mathcal M$, see [6]; in particular, $\nu (\mathcal M')=0$. Therefore, it suffices to prove the proposition for $\nu$-a.e. $x\in \mathcal M\setminus \mathcal M'$. Let $\widetilde{\mathcal M}$ denote the lift of $\mathcal M\setminus \mathcal M'$ to $\Omega \mathcal T_g$.

Fix $\phi \in \widehat{{\,\mathrm{Mod}\,}}(S_g)$ (that is, $\phi$ is not torsion). Define

$$\begin{aligned} P(\phi )= \Big \{ x \in \widetilde{\mathcal M}: \quad A \cdot x = \phi (x), \quad \text {for some}\quad A \in \mathrm{{SL}}_2(\mathbb R) \Big \}. \end{aligned}$$

We will show, for every $\phi \in \widehat{{\,\mathrm{Mod}\,}}(S)$, $P(\phi )$ is a $\nu$–measure zero subset of $\widetilde{\mathcal M}$. Note that, by assumption, $\dim (\widetilde{\mathcal M}) >3$.

Consider $x \in P(\phi )$ and let $E_x$ be the $\mathrm{{GL}}^+(2, \mathbb {R})$ orbit or *x*. Then $E_x$ can be considered as (an open subset of) the tangent space of the Teichmüller disk $\mathbb {H}_x$ associated to *x* (the projection of $E_x$ to Teichmüller space). The restriction of Teichmüller metric to $\mathbb {H}_x$ equips $\mathbb {H}_x$ with the hyperbolic metric (up to a factor 2). We observe that $\phi$ stabilizes $\mathbb {H}_x$ acting on $\mathbb {H}_x$ by an isometry. In fact, we have either (see, for example, [11, Lemma 5.6])

- $\phi$ acts loxodromically on $\mathbb {H}_x$ and $\phi$ a pseudo-Anosov element.
- $\phi$ acts parabolically on $\mathbb {H}_x$ and $\phi$ is a multi-curve.
- $\phi$ acts elliptically on $\mathbb {H}_x$ and $\phi$ has finite order in ${{\,\mathrm{Mod}\,}}(S)$.

Note that the third case is excluded since we are assuming $\phi$ is not torsion. We argue each case separately showing that $P(\phi )\cap \widetilde{\mathcal M}$ is a $\nu$-measure zero subset of $\widetilde{\mathcal M}$.

#### Remark 3.3

We are in fact proving more that what is stated in Proposition 3.2. Recall that, for $x \in \widetilde{\mathcal M}$, the Veech group of *x* is the subgroup of $\mathrm{PSL}_2(\mathbb {R})$ which stabilizes $\mathbb H_x$ setwise. Hence, the proof actually gives that for $\nu$-a.e. $x\in \widetilde{\mathcal M}$, the Veech group of *x* is finite.

### $\phi$ is pseudo-Anosov element

A pseudo-Anosov map $\phi$ stabilizes only one Teichmüller disk, the one where $\partial \mathbb {H}_x$ contains $F_+(\phi )$ and $F_-(\phi )$; the stable and the unstable foliation associated to $\phi$. Therefore, $P(\phi )= T_1 \mathbb {H}_x$, the unit tangent bundle over $\mathbb {H}_x$. Since $\mathcal M$ is a not a Teichmüller curve, it has a dimension larger than 3. Hence $P(\phi )\cap \widetilde{\mathcal M}$ is a $\nu$-measure zero subset of $\widetilde{\mathcal M}$.

### $\phi$ is a multi-twist

Let $\phi$ be a multi-twist around $\gamma$, namely

$$\begin{aligned} \phi = \prod D_{\gamma _i}^{p_i}. \end{aligned}$$

Let ${\mathbb {R}}P(\phi )$ be the subset of $\mathcal H(\alpha )$ obtained from points in $P(\phi )$ after scaling. Then, for any $x \in {\mathbb {R}}P(\phi )$, a measured foliation that is topologically equivalent to $\gamma = \{ \gamma _1, \dots , \gamma _k)$ has to appear in the boundary of $\mathbb {H}_x$. That is, after a rotation, we can assume $x= (F_-, F_+)$ and $F_+ = \sum c_k \gamma _k$. Furthermore, *x* has a cylinder decomposition where the modulus of these cylinders are rationally multiples of each other ([11, Lemma 5.6]). That is, there are $r_i \in {\mathbb Q}$ such that

$$\begin{aligned} r_i \cdot \frac{i(F_-, \gamma _i)}{c_i} = r_j \cdot \frac{i(F_-, \gamma _j)}{c_j}, \end{aligned}$$

for $1 \le i, j \le k$. We also have

$$\begin{aligned} \sum c_i \cdot i(F_-, \gamma _i)={{\,\mathrm{area}\,}}(x). \end{aligned}$$

That is, given $\gamma$, $r_i$, $F_-$ and ${{\,\mathrm{area}\,}}(x)$, we can calculate the values of $c_i$. Hence, $F_+$ and subsequently *x* are uniquely determined by $\gamma$, $r_i$, $F_-$ and ${{\,\mathrm{area}\,}}(x)$. There are countably many choices for the values $r_i$ and the multi-curve $\gamma$. We now show that the dimension of the space of possible measured foliations $F_-$ is half the dimension of ${\mathbb {R}}\widetilde{\mathcal M}$ where ${\mathbb {R}}\widetilde{\mathcal M}$ is the subset of $\mathcal H(\alpha )$ obtained from point in $\widetilde{\mathcal M}$ after scaling.

For a filling bi-recurrent train-track $\tau$ (see [15] for definition and discussion) any admissible weight on $\tau$ defines a measured foliation. We then say this measured foliation is carried by $\tau$. The complementary regions of a filling train tracks are *n*–gons or punctured *n*–gons. A foliation carried by $\tau$ has a singular point associated to each complementary region of $\tau$. We say $\tau$ is of type $\alpha =(\alpha _1,\ldots ,\alpha _{m})$ if $\tau$ has *m* complementary components that are punctured $\alpha _i$–gons, $i = 1 \dots m$. We denote the space of admissible weights in $\tau$ by $W(\tau )$.

#### Lemma 3.4

For every $x \in \mathcal H(\alpha )$ there are train tracks $\tau _+$ and $\tau _-$ of type $\alpha$ such that a neighborhood of $\mathcal H(\alpha )$ around *x* is homeomorphic to $U \times V$ where *U*, *V* are open subsets of $W(\tau _+)$ and $W(\tau _-)$ respectively. In fact, the real part of the period coordinates for $\mathcal H(\alpha )$ give coordinates for *U* and the imaginary part of the period coordinates, give coordinates for *V*.

#### Proof

Let $\Delta$ be a triangulation of *x* by saddle connections (for example, $L^\infty$-Delanay triangulations see [7, §3]). Pick a subset $\mathcal B$ of the edges of $\Delta$ that give a basis for the homology of *x* relative to the zeros $\Sigma$ of *x*. Then the complex numbers $\{ \int _\omega x \} _{\omega \in \mathcal B}$ give local coordinates for $H(\alpha )$. For every edge $\omega$ of $\Delta$, we have

$$\begin{aligned} \text{ i }(\omega , F_-)= \mathfrak {R}\left( \int _\omega x\right) . \end{aligned}$$

In fact, $F_-$ can be constructed, triangle by triangle, from the set of real numbers $\{\text{ i }(\omega , F_-) \}_{\omega \in \Delta }$. That is there is a train-track $\tau _-$ dual to the triangulation $\Delta$ (again, see [7, §3] for the construction of such train-tracks) such that $\{ \mathfrak {R}( \int _\omega x ) \} _{\omega \in \mathcal B}$ form an admissible weights on $\tau _-$. At any point $y \in \mathcal H(\alpha )$ near *x*, the triangulation $\Delta$ can still be represented by saddle connections and the set $\{ \mathfrak {R}( \int _\omega y ) \} _{\omega \in \mathcal B}$ form an admissible weights on $\tau _-$ that is associated to the vertical foliation at *y*. That is, $\{ \mathfrak {R}( \int _\omega y ) \} _{\omega \in \mathcal B}$, thought of as admissible weights on $\tau _-$ give local cooridinates for the set of measured foliation that appear as a horizontal foliation of an element of $\mathcal H_1(\alpha )$ near *x*. The same also holds for $\tau _+$ and the vertical foliations. $\square$

Since ${\mathbb {R}}\widetilde{\mathcal M}$ is an affine sub-manifold of $\mathcal H(\alpha )$, it is locally defined by a set of affine equations on period coordinates, see e.g. §3.1 and [5]. That is, there are subspaces $U' \subset U$ and $V' \subset V$, defined by the same set of affine equations, such that a neighborhood of *x* in ${\mathbb {R}}\widetilde{\mathcal M}$ is naturally homeomorphic to $U' \times V'$. In particular, where $U'$ and $V'$ have half the dimension of ${\mathbb {R}}\widetilde{\mathcal M}$.

Let *W* be the intersection of ${\mathbb {R}}P(\phi )$ with this neighborhood. Recall that, fixing the multi-curve $\gamma$, rational numbers $r_i$ and the area, every point in *W* is determined, up to rotation, by a point in $U'$. Therefore, *W* is a countable union of set of dimension $\dim (U') +2$. But

$$\begin{aligned} \dim (U') +2= \frac{1}{2} \dim ({\mathbb {R}}\widetilde{\mathcal M}) +2 < \dim ({\mathbb {R}}\widetilde{\mathcal M}), \end{aligned}$$

where the last inequality follows from the assumption that $\dim ({\mathbb {R}}\widetilde{\mathcal M}) >4$. That is, ${\mathbb {R}}P(\phi )\cap {\mathbb {R}}\widetilde{\mathcal M}$ is a countable union of lower dimensional subset of ${\mathbb {R}}\widetilde{\mathcal M}$ and therefore, has $\bar{\nu }$-measure zero, see §3.1 for the definition of $\bar{\nu }$. Since, $\mathrm{Stab}_{\mathrm{{SL}}_2(\mathbb R)}(x)$ does not change after scaling, we have, $P(\phi )\cap \widetilde{\mathcal M}$ has $\nu$–measure zero in $\widetilde{\mathcal M}$.

### Proof of Theorem 3.1

In this section we prove Theorem 3.1. The proof is based on the following proposition.

#### Proposition 3.5

Let $\{E_k: k\in \mathbb {N}\}\subset \mathcal H_1(\alpha ) \subset \Omega \widehat{\mathcal M}_{g,n}$ be a sequence of closed $\mathrm{{SL}}_2(\mathbb {R})$ orbits each equipped with the $\mathrm{{SL}}_2(\mathbb {R})$-invariant probability measure $\mu _k$. Assume further that there exists an affine invariant submanifold $(\mathcal M,\nu )\subset \mathcal H_1(\alpha )$ so that

3.3 $$\begin{aligned} \mu _k\rightarrow \nu \quad \text {as }\quad k\rightarrow \infty . \end{aligned}$$

Let $V_k$ denote the Teichmüller curve associated to $E_k$ for all *k*. Then $\{V_k\}$ Benjamini-Schramm converges to $\mathbb {H}$.

#### Proof

The proof if based on Proposition 1.1. Let us write $E_k=\mathrm{{SL}}_2(\mathbb {R}).x_k$. We will show that $\mathrm{{SL}}_2(\mathbb {R})/\mathrm{Stab}_{\mathrm{{SL}}_2(\mathbb {R})}(x_k)$ Benjamini-Schramm converges to $\mathrm{{SL}}_2(\mathbb {R})$ from which the proposition follows.

First note that $(\mathcal M,\nu )$ is not a closed $\mathrm{{SL}}_2(\mathbb {R})$ orbits, see [6, Thm. 2.3]. Hence, by Proposition 3.2, we have

3.4 $$\begin{aligned} \mathrm{Stab}_{\mathrm{{SL}}_2(\mathbb R)}(x)=\{e\}\text { for } \nu -\text {a.e.} \quad x\in \mathcal M. \end{aligned}$$

In the remaining pats of the argument, we write $H=\mathrm{{SL}}_2(\mathbb {R})$ and use the notation in §1. In particular, for all $R>0$, let

$$\begin{aligned} B^H(e,R)=\{h\in H: \Vert h-I\Vert< R\text { and }\Vert h^{-1}-I\Vert < R\} \end{aligned}$$

where $\Vert \;\Vert$ denotes the maximum norm on $\mathrm{Mat}_2(\mathbb {R})$ with respect to the standard basis. Similarly, for $r>0$, let $B^H_\mathrm{Rie}(e,r)$ denote the ball of radius *r* centered at the identity with respect the bi-$\mathrm{SO}(2)$-invariant Riemannian metric on *H* induced using the Killing form.

For every $x\in \mathcal H_1(\alpha )$, let $r_x$ denote 1/2 of the injectivity radius of *x* in $\mathcal H_1(\alpha )$ with respect to the Teichmüller metric. Then $x\mapsto r_x$ is continuous on $\mathcal H_1(\alpha )$; moreover, $h\mapsto hx$ is injective on $B^H_\mathrm{Rie}(e,r_x)$.

Let $R>0$ and for every $x\in \mathcal M$, put $B^H_R(x):= \bar{B}^H(e,R)\setminus B^H_\mathrm{Rie}(e,r_x)$; note that this a compact subset of $\mathrm{{SL}}_2(\mathbb {R})$. Define $f_R:\mathcal M\rightarrow [0,\infty )$ by

$$\begin{aligned} f_R(x)=\min \Bigl \{\mathrm{dist}_{\mathrm{Teich}}(x,hx): h\in B^H_R(x)\Bigr \}. \end{aligned}$$

Note that $f_R$ is continuous. Indeed, let $y_m\rightarrow y$, and let $h_m\in B^H_R(y_m)$ be so that $f_R(y_m)=\mathrm{dist}_{\mathrm{Teich}}(y,h_my_m)$. Let $\{f_R(y_{m_i})\}$ be a converging subsequence of $\{f_R(y_m)\}$. Since $B^H_R(y_m)$ converges to $B^H_R(y)$ (in Hausdorff metric on compact sets), there is a subsequence $h_{m_{i_j}}\rightarrow h\in B^H_R(y)$ which implies: $f_R(y)\le \lim _i f_R(y_{m_i})$. In consequence, $f_R(y)\le \liminf f_R(y_m)$. To see the opposite direction, let $h\in B^H_R(y)$ be so that $f_R(y)=\mathrm{dist}_{\mathrm{Teich}}(y,hy)$. Let $h_m\in B^H_R(y_m)$ be so that $h_m\rightarrow h$, then $f_R(y_m)\le \mathrm{dist}_{\mathrm{Teich}}(y,h_my_m)$ and for every $\varepsilon >0$ we have $\mathrm{dist}_{\mathrm{Teich}}(y,h_my_m)\le \mathrm{dist}_{\mathrm{Teich}}(y,hy)+\varepsilon =f_R(y)+\varepsilon$ so long as *m* is large enough. Hence $\limsup f_R(y_m)\le f_R(y)+\varepsilon$. The continuity of $f_R$ follows.

Moreover, in view of (3.4), we have $f_R(x)>0$ for $\nu$-a.e. $x\in \mathcal M$. Finally, since for every *x*, the map $h\mapsto hx$ is injective on $B^H_\mathrm{Rie}(e,r_x)$, we have $\mathrm{Stab}_{\mathrm{{SL}}_2(\mathbb R)}(x)\cap B^H_\mathrm{Rie}(e,r_x)=\{e\}$. Thus if $f_R(x)>0$ for some $x\in \mathcal M$, then $\mathrm{Stab}_{\mathrm{{SL}}_2(\mathbb R)}(x)\cap B^H(e,R)=\{e\}$.

Altogether, we deduce that $f_R$ satisfies the conditions in Proposition 1.1. This and (3.3) imply that Proposition 1.1 applies and yields:

$\mathrm{{SL}}_2(\mathbb {R})/\mathrm{Stab}_{\mathrm{{SL}}_2(\mathbb {R})}(x_k)$ Benjamini-Schramm converges to $\mathrm{{SL}}_2(\mathbb {R})$.

The proof is complete. $\square$

#### Proof of Theorem 3.1

Let $\{V_k: k\in \mathbb {N}\}\subset \widehat{\mathcal M}_{g,n}$ be a sequence of Teichmüller curves. We will show that for every subsequence $\{V_{k_i}\}$, there exists a further subsequence $\{V_{k_{i_j}}\}$ which Benjamini-Schramm converges to $\mathbb {H}$ the theorem follows from this.

Let $\{V_{k_i}\}$ be a subsequence of $\{V_k\}$. Passing to a further subsequence, which we continue to denote by $\{V_{k_i}\}$, we may assume that the corresponding $\mathrm{{SL}}_2(\mathbb {R})$ orbits $\{E_{k_i}\}$ lie in $\mathcal H_1(\alpha )\subset \Omega \widehat{\mathcal M}_{g,n}$ for a fixed $\alpha$.

Now by [6, Thm. 2.3], see also [6, Cor. 2.5], there exists a subsequence $\{E_{k_{i_j}}\}$ of $\{E_{k_i}\}$, and an affine invariant manifold $(\mathcal M, \nu )$, so that $\mu _{k_{i_j}}\rightarrow \nu$ where $\mu _{k_{i_j}}$ denotes the $\mathrm{{SL}}_2(\mathbb {R})$-invariant measure on $E_{k_{i_j}}$.

By Proposition 3.5, we have $V_{k_{i_j}}$ Benjamini-Schramm converges to $\mathbb {H}$; as we wished to show. $\square$

## Data Availability

No datasets were generated or analyzed during the current study.

## Footnotes

1 The works [2, 3] are indeed more general and allow for properly immersed maximal totally geodesic submanifolds of dimension at least 2.
